# Prognostic Value of Early Nerve Conduction Studies in Suspected Guillain-Barré Syndrome in Pediatric Age Group: An Observational Study

**DOI:** 10.7759/cureus.71683

**Published:** 2024-10-17

**Authors:** Om Prakash, Yogesh Kumar, Tribhuwan Kumar, Lokesh K Tiwari

**Affiliations:** 1 Physiology, All India Institute of Medical Sciences, Patna, Patna, IND; 2 Pediatrics, All India Institute of Medical Sciences, Patna, Patna, IND; 3 Pediatrics, All India Institute of Medical Sciences, Rishikesh, Rishikesh, IND

**Keywords:** aidp, aman, guillain–barré syndrome, nerve conduction studies, pediatric, prognosis

## Abstract

Guillain-Barré syndrome (GBS) is a rare but potentially life-threatening autoimmune disorder affecting the peripheral nervous system. In pediatric patients, early diagnosis and intervention are crucial for improved outcomes. This observational study aimed to evaluate the prognostic value of early nerve conduction studies (NCS) in pediatric patients diagnosed with GBS. The study was conducted at All India Institute of Medical Sciences (AIIMS) Patna from January 2022 to March 2023, involving 11 patients aged between five and 14 years. All patients underwent NCS on three occasions: within three days, between three to seven days, and between 7 and 14 days after admission. The findings indicated that 63.64% of the patients exhibited a demyelinating pattern (acute inflammatory demyelinating polyneuropathy, AIDP), while 36.36% showed an axonal pattern (acute motor axonal neuropathy, AMAN). Early NCS findings were critical in predicting the severity and guiding the timely initiation of appropriate treatment, leading to improved outcomes. This study underscores the importance of early electrophysiological assessment in pediatric GBS for optimal patient management and prognosis.

## Introduction

Guillain-Barré syndrome (GBS) is an acute, immune-mediated polyneuropathy characterized by rapidly progressive, symmetrical, ascending limb weakness and areflexia [1]. It is the most common cause of acute flaccid paralysis in children and adults, with an estimated annual incidence of 0.6 to 2.4 cases per 100,000 person-years [2]. The condition typically follows a viral or bacterial infection, with *Campylobacter jejuni* being the most commonly identified preceding pathogen [3].

In pediatric patients, GBS presents unique challenges due to its variable clinical presentation and the potential for rapid progression to respiratory failure [4]. Early diagnosis is crucial for initiating appropriate treatment and preventing complications. However, the initial symptoms of GBS in children can be subtle and may mimic other neurological disorders, making prompt and accurate diagnosis challenging [5].

Nerve conduction studies (NCS) have emerged as a valuable tool in the diagnosis and classification of GBS [6]. These electrophysiological tests can detect abnormalities in peripheral nerve function before clinical signs become apparent, potentially allowing for earlier intervention [7]. Moreover, NCS can help differentiate between the main subtypes of GBS: acute inflammatory demyelinating polyneuropathy (AIDP), which affects the myelin sheath, and the axonal forms, including acute motor axonal neuropathy (AMAN), and acute motor-sensory axonal neuropathy (AMSAN) [8].

While the utility of NCS in adult GBS patients is well-established, less is known about its prognostic value in pediatric cases [9]. This study aims to bridge this knowledge gap by exploring the role of early NCS in predicting outcomes for children with GBS. By correlating electrophysiological findings with clinical progression and recovery, we seek to enhance our understanding of pediatric GBS and improve patient care.

The primary objective of this study was to evaluate the prognostic value of early NCS in pediatric GBS patients, focusing on their ability to predict disease severity and guide treatment decisions. Secondary objectives included characterizing the clinical and demographic profile of pediatric GBS patients, assessing the frequency of nerve involvement, and classifying patients into GBS subtypes based on NCS findings.

This research is particularly relevant given the potential for severe outcomes in pediatric GBS, including respiratory failure and long-term disability [10]. By identifying early electrophysiological markers of disease severity and progression, clinicians may be better equipped to tailor treatment strategies and improve patient outcomes.

## Materials and methods

Study design and setting

The protocol of the study was approved by the Institutional Research Cell (Ref. No. AIIMS/Pat/IRC/2020/PGTh/Jan21/24) and Institutional Ethics Committee of All India Institute of Medical Sciences (AIIMS) Patna (Ref. No. AIIMS/Pat/IEC/PGTh/Jan21/24). Informed written consent was obtained from parents of all enrolled children. This prospective observational study was conducted from January 2022 to March 2023 at AIIMS Patna, a tertiary care center in Bihar, India.

Participants

The study included pediatric patients diagnosed with GBS based on Asbury's criteria [11]. The inclusion criteria for the study were participants aged one to 15 years, presenting with ascending areflexic quadriparesis evolving over up to four weeks, and without other identifiable causes of acute polyneuropathy. However, the study population ultimately ranged from five to 14 years. Patients with chronic polyneuropathy, upper motor neuron signs, significant pulmonary disease, or severe electrolyte imbalances affecting nerve conduction studies were excluded.

This prospective observational study was conducted over a 15-month period at the pediatric inpatient department of AIIMS Patna. A total of 11 patients meeting the predefined inclusion criteria were enrolled in the study. GBS is relatively uncommon in the pediatric population, with an estimated annual incidence of 0.4 to 1.3 cases per 100,000 children. While this sample size is small, it still provides valuable insights into the electrophysiological patterns and clinical characteristics of pediatric GBS in our setting. Future studies with longer enrollment periods or multi-center collaborations could build upon these findings with larger sample sizes.

Demographic data, clinical history, and examination findings were recorded for all patients at admission. Assessments included motor power using the Medical Research Council (MRC) sum score [12], deep tendon reflexes, sensory symptoms, and autonomic dysfunction. The Hughes Functional Grading Scale (FGS) was used to assess disease severity and monitor progress [13].

NCS were performed using a Neurosoft Neuro MEP-NET EMG/NCS/EP system (Neurosoft, Ivanovo, Russia) on three occasions: within three days of admission, between three to seven days after admission, and between seven and 14 days after admission. Median, ulnar, and peroneal nerves were tested during motor study. Median, ulnar, and sural nerves were tested for sensory study. F waves were checked for median and peroneal nerve. Based on the findings, the test subjects were classified into AMAN type or AIDP type. Table 1 summarizes the parameters assessed and the electrophysiological classification criteria [14].

**Table 1:** Electrophysiological classification criteria.

Electrodiagnostic parameter	AMAN (acute motor axonal neuropathy)	AIDP (acute inflammatory demyelinating polyneuropathy)
Motor nerve conduction velocity	Normal or slightly reduced	Markedly reduced
Distal motor latency	Normal or slightly prolonged	Significantly prolonged
F-wave latency	Normal or slightly prolonged	Significantly prolonged or absent
Compound muscle action potential (CMAP) amplitude	Reduced	Normal in early stages, may reduce later
Sensory nerve action potential (SNAP)	Normal	Reduced or absent
Conduction block	Absent	Present
Temporal dispersion	Absent	Present
Sural sparing pattern	Absent	May be present

Patients were classified into GBS subtypes based on these criteria, allowing for a comprehensive electrophysiological assessment of their condition.

Treatment and follow-up

All patients received immunotherapy, with intravenous immunoglobulin (IVIg) as the first-line treatment (2 g/kg divided over two to five days). For patients showing inadequate improvement (defined as less than one-grade improvement on the Hughes Functional Grading Scale {FGS}) after seven to 10 days, a second course of IVIg was considered. Treatment failure was defined as no improvement or worsening in Hughes FGS score after two full courses of IVIg, or progression to mechanical ventilation despite immunotherapy.

Plasmapheresis was used in combination with IVIg for two patients with rapidly progressive weakness or those failing to respond to IVIg alone. Intensive physiotherapy was initiated early and continued throughout the hospital stay, tailored to each patient's functional status. Pain management included gabapentin or pregabalin for neuropathic pain, with opioids reserved for severe cases. Autonomic dysfunction, when present, was carefully monitored and managed, with particular attention to cardiac arrhythmias, blood pressure fluctuations, and gastrointestinal motility issues.

Patients were followed up until discharge, with the Hughes FGS used to assess functional outcomes at regular intervals and at discharge.

Statistical analysis

Data were analyzed using IBM SPSS Statistics for Windows, version 25 (IBM Corp., Armonk, NY, USA). Descriptive statistics were used for demographic and clinical characteristics. The Chi-square test was used for categorical variables, and the Student's t-test or Mann-Whitney U test for continuous variables, as appropriate. Correlations between NCS parameters and clinical outcomes were assessed using Pearson's or Spearman's correlation coefficients. A p-value <0.05 was considered statistically significant.

## Results

Demographic and clinical characteristics

The study cohort included 11 pediatric patients diagnosed with GBS, consisting of six males (54.55%) and five females (45.45%). Table 2 provides a summary of the demographic and clinical characteristics of the participants. Antecedent infections were observed in both the respiratory and gastrointestinal systems. Among respiratory infections, *Mycoplasma pneumoniae* was the most frequently identified pathogen, while *Campylobacter jejuni* was the predominant pathogen associated with gastrointestinal infections.

**Table 2:** Demographic and clinical characteristics of pediatric GBS patients. CSF: cerebrospinal fluid; GBS: Guillain-Barré syndrome.

Characteristic	Value
Total patients	11
Gender (male:female)	6:5
Mean age (years)	9 ± 3.26
Age range (years)	5-14
Preceding infection	8 (72.73%)
Mean time to admission (days)	5.36 ± 2.94
Motor weakness	11 (100%)
Sensory symptoms	7 (63.64%)
Cranial nerve involvement	4 (36.36%)
Autonomic dysfunction	3 (27.27%)
Antecedent respiratory involvement	5 (45.45%)
Antecedent gastrointestinal infections	3 (27.27%)
Cytoalbuminologic dissociation in CSF	7 (63.64%)

Electrophysiological findings

The electrophysiological findings among the participants demonstrated varying degrees of motor and sensory involvement. These results are summarized in Table 3, detailing the nerve conduction studies, including latency, amplitude, and conduction velocity across affected nerves.

**Table 3:** Summary of nerve conduction study findings. CMAP: compound muscle action potential; DML: distal motor latency; MCV: motor conduction velocity.

Parameter	AIDP (n=7)	AMAN (n=4)	p-value
Median nerve CMAP amplitude (mV)	2.8 ± 1.4	1.9 ± 0.8	0.039
Median nerve DML (ms)	5.2 ± 1.1	3.8 ± 0.6	0.027
Median nerve MCV (m/s)	32.4 ± 6.7	48.2 ± 5.3	0.005
Ulnar nerve CMAP amplitude (mV)	3.1 ± 1.6	2.3 ± 1.1	0.048
Ulnar nerve DML (ms)	4.8 ± 0.9	3.5 ± 0.5	0.031
Ulnar nerve MCV (m/s)	34.6 ± 7.1	50.1 ± 4.8	0.003
Peroneal nerve CMAP amplitude (mV)	1.6 ± 0.9	0.8 ± 0.5	0.021
Peroneal nerve DML (ms)	6.4 ± 1.3	4.2 ± 0.7	0.018
Peroneal nerve MCV (m/s)	28.7 ± 5.9	43.6 ± 4.2	0.002
F-wave abnormalities	7 (100%)	1 (25%)	<0.001
Sensory nerve abnormalities	6 (85.71%)	0 (0%)	<0.001

Clinical course and outcomes

The mean time to peak disability, defined as the highest Hughes FGS score, was 15.18 ± 7.07 days, ranging from seven to 22 days. Table 4 presents the clinical course and outcomes for the study cohort.

**Table 4:** Clinical course and outcomes of pediatric GBS patients. FGS: Functional Grading Scale; ICU: intensive care unit; GBS: Guillain-Barré syndrome.

Parameter	Value
Mean time to peak disability (days)	15.18 ± 7.07
Range of time to peak disability (days)	7-22
Mean Hughes FGS at admission	3.45 ± 0.82
Mean Hughes FGS at nadir	4.18 ± 0.98
Mean Hughes FGS at discharge	2.36 ± 0.81
Patients requiring ICU admission	3 (27.27%)
Patients requiring mechanical ventilation	2 (18.18%)
Mean length of hospital stay (days)	18.64 ± 6.73
Patients with complete recovery at discharge	4 (36.36%)
Patients with residual deficits at discharge	7 (63.64%)

Correlation of NCS findings with clinical outcomes

Early NCS findings, particularly those obtained within the first three days of admission, showed significant correlations with clinical outcomes. Table 5 summarizes these correlations.

**Table 5:** Correlation of early NCS findings with clinical outcomes. CMAP: compound muscle action potential; MCV: motor conduction velocity; NCS: nerve conduction studies.

NCS parameter	Correlation with peak disability	Correlation with recovery time
Median nerve CMAP amplitude	r = -0.72, p = 0.013	r = -0.68, p = 0.021
Peroneal nerve CMAP amplitude	r = -0.79, p = 0.004	r = -0.74, p = 0.009
Median nerve MCV	r = -0.65, p = 0.031	r = -0.61, p = 0.046
F-wave abnormalities	r = 0.70, p = 0.017	r = 0.67, p = 0.024

The results indicate that early reductions in compound muscle action potential (CMAP) amplitudes, particularly in the median and peroneal nerves, were strongly correlated with greater peak disability (higher Hughes FGS scores) and longer recovery times. Decreased motor conduction velocities in the median nerve also showed a significant correlation with worse clinical outcomes, though to a lesser extent than CMAP amplitudes. The presence of F-wave abnormalities in early NCS was positively correlated with both peak disability and recovery time, suggesting that these abnormalities may be indicative of more severe disease.

Subgroup analysis: AIDP vs. AMAN

Although several differences in the clinical course and outcomes between the AIDP and AMAN subgroups were observed, only the time to peak disability was statistically significant (Table 6). Other outcomes showed trends but did not reach statistical significance.

**Table 6:** Comparison of clinical course and outcomes between AIDP and AMAN subgroups. AIDP: acute inflammatory demyelinating polyneuropathy; AMAN: acute motor axonal neuropathy; ICU: intensive care unit.

Parameter	AIDP (n=7)	AMAN (n=4)	p-value
Time to peak disability (days)	17.3 ± 6.2	11.5 ± 4.8	0.041
ICU admission	2 (28.57%)	1 (25%)	0.892
Complete recovery at discharge	2 (28.57%)	2 (50%)	0.480
Residual deficits at discharge	5 (71.43%)	2 (50%)	0.480

## Discussion

This prospective observational study provides valuable insights into the prognostic value of early nerve conduction studies in pediatric patients with GBS. The findings underscore the importance of timely electrophysiological assessment in predicting disease severity and guiding clinical management.

Demographic and clinical profile

The demographic profile of our cohort aligns with previous reports on pediatric GBS, with a slight male predominance and a mean age of nine years [15]. The high prevalence of preceding infections in eight (72.73%) patients supports the established association between GBS and antecedent immune triggers [16]. The variability in clinical presentations, including sensory symptoms, cranial nerve involvement, and autonomic dysfunction, highlights the heterogeneous nature of pediatric GBS and the diagnostic challenges it presents [17].

Electrophysiological patterns and subtypes

Our study found a higher prevalence of AIDP (n=7, 63.64%) compared to AMAN (n=4, 36.36%), which is consistent with the pattern typically observed in Western countries and parts of India [18]. However, this distribution differs from some East Asian countries where AMAN is more prevalent [19]. This variation underscores the importance of regional epidemiological studies in understanding GBS patterns.

The distinct electrophysiological features of AIDP and AMAN observed in our study corroborate previous findings [20]. AIDP patients exhibited classical signs of demyelination, including prolonged distal motor latencies, reduced conduction velocities, and frequent F-wave abnormalities. In contrast, AMAN patients showed primarily axonal damage, manifested as reduced CMAP amplitudes with relatively preserved conduction velocities. These distinctions are crucial for accurate subtype classification and have implications for prognosis and treatment [21].

Prognostic value of early NCS

One of the key findings of our study is the significant correlation between early NCS parameters and clinical outcomes. The strong negative correlation between CMAP amplitudes (particularly in median and peroneal nerves) and both peak disability and recovery time suggests that early axonal loss or conduction block may be predictive of a more severe disease course. This aligns with previous studies in adult populations, which have shown that reduced CMAP amplitudes in the early stages of GBS are associated with poorer outcomes [22].

The correlation between F-wave abnormalities and worse clinical outcomes is particularly noteworthy. F-waves reflect the excitability of motor neurons and can be affected early in the disease process, even before significant changes in distal nerve segments [23]. Our findings suggest that F-wave abnormalities in early NCS may serve as a valuable prognostic indicator in pediatric GBS.

Clinical implications of electrophysiological subtypes

The observed differences in clinical course between AIDP and AMAN subtypes, although not statistically significant due to our small sample size, warrant further investigation. The tendency for AIDP patients to reach peak disability later than AMAN patients is consistent with some previous reports [24]. This could be related to the different pathophysiological mechanisms: AIDP typically involves a more gradual process of demyelination, while AMAN can cause rapid axonal degeneration [25].

The similar rates of ICU admission between AIDP and AMAN patients in our cohort suggest that both subtypes can lead to severe presentations requiring intensive care. However, the trend towards better recovery rates at discharge for AMAN patients is intriguing and contrasts with some studies that have reported poorer short-term outcomes for AMAN [26]. This discrepancy might be due to our small sample size or could reflect the benefits of early aggressive treatment in our cohort.

Therapeutic implications

The prognostic information derived from early NCS has important implications for therapeutic decision-making. Patients with severe electrophysiological abnormalities, particularly marked reductions in CMAP amplitudes or widespread F-wave abnormalities, may benefit from more aggressive or combined immunotherapies [27]. In our study, two patients with rapid progression and severe NCS abnormalities received combination therapy with IVIg and plasmapheresis, highlighting the role of electrophysiological findings in guiding treatment intensification.

Moreover, the ability to predict a more prolonged recovery based on early NCS findings can inform patient and family counseling, resource allocation, and rehabilitation planning. This is particularly crucial in pediatric patients, where prolonged hospitalization and rehabilitation can have significant impacts on education and psychosocial development [28].

Limitations and future directions

Our study has several limitations that should be considered. The small sample size limits the statistical power and generalizability of our findings. A larger, multi-center study would be valuable to confirm these results and potentially identify more subtle prognostic indicators. Additionally, while we followed patients until discharge, longer-term follow-up would provide insights into the correlation between early NCS findings and long-term functional outcomes.

Although the H-reflex has been shown to be sensitive in detecting early phases of GBS, it was not included in this study due to logistical constraints. Future studies should consider incorporating H-reflex measurements for a more comprehensive evaluation of early electrodiagnostic abnormalities in GBS.

The lack of systematic autoantibody testing is a limitation of this study. Future research should aim to incorporate comprehensive autoantibody profiling, including but not limited to anti-GM1, anti-GQ1b, and other relevant antibodies. This would enable a more detailed analysis of the relationship between specific autoantibodies and GBS development, clinical course, and treatment outcomes in pediatric patients.

Future research could explore the integration of early NCS findings with other prognostic factors, such as clinical features, biomarkers, and neuroimaging, to develop a comprehensive prognostic model for pediatric GBS. Furthermore, serial NCS throughout the disease course could provide valuable information on the evolution of electrophysiological abnormalities and their relationship to clinical recovery.

## Conclusions

This study demonstrates the significant prognostic value of early nerve conduction studies in pediatric patients with GBS. Early reductions in CMAP amplitudes and the presence of F-wave abnormalities are strongly correlated with greater peak disability and longer recovery times. The electrophysiological distinction between AIDP and AMAN subtypes provides insights into the pathophysiological mechanisms and may have implications for clinical course and management.

These findings underscore the importance of performing NCS early in the course of pediatric GBS. The prognostic information derived from these studies can guide clinical decision-making, inform treatment strategies, and assist in counseling patients and families about expected outcomes. While larger studies are needed to validate and extend these findings, our results suggest that early NCS should be considered an essential component of the diagnostic and prognostic workup for pediatric GBS patients.
